# Supplementary material for: The restoration of the endangered Sambucus palmensis after 30 years of conservation actions in the Garajonay National Park: genetic assessment and niche modeling
Source: PeerJ. 2018 Jun 12;6:e4985. doi: 10.7717/peerj.4985 (PMC6003393; doi:10.7717/peerj.4985)
Supplement: Supplemental Information 1 — EMBL ID: Accession number per locus. Dye: Name of each fluorescent dye per locus. Multiplex; ‡, Load A; *, Load B; F, Forward; R, Reverse. [file peerj-06-4985-s001.docx]

**The restoration of the endangered *Sambucus palmensis* after 30 years of conservation actions in the Garajonay National Park: genetic assessment and niche modelling**

**P. Rodríguez-Rodríguez^1^, A. G. Fernández de Castro^2^, P.A. Sosa^1^**

1. Instituto Universitario de Estudios Ambientales y Recursos Naturales (IUNAT), Universidad de Las Palmas de Gran Canaria, Campus Universitario de Tafira, 35017 Las Palmas de Gran Canaria, España.

| Locus | EMBL ID | Motif | PCR primer sequence (5’ -> 3’) | Size Range (bp) | Dye |
| --- | --- | --- | --- | --- | --- |
| EMSn017 | AM086423 | (AG)21 – (AG)24 | F: GGTATTGCTTGAACAATCATCG | 202-206 | 6-FAM ‡ |
|  |  |  | R: GCCTTTTGCCCAAACTATCC |  |  |
| EMSn025 | AM086426 | (AC)13 – (AC)8 | F: AATGCATCGCAAGAAAAAGG | 191-197 | 6-FAM * |
|  |  |  | R: GGTAAGATAAATGATACAATGTTTTGG |  |  |
| EMSn003 | AM086420 | (CT)11 | F: TCGTCTTTTCCGACTCTAAAGC | 202-224 | NED * |
|  |  |  | R: CTGGACATTTGCGATCTGG |  |  |
| Sam_Tet2 | LT600693 | (AGAT)12 | F: AAATGCACTGAACAGTGGTTGA | 118-146 | VIC ‡ |
|  |  |  | R: CCCTAGTCCTCCAACCCATC |  |  |
| Sam_Hex2 | LT600694 | (AATAAC)6 | F: TGATGATGGTTGTTGGTAGATCA | 218-224 | PET ‡ |
|  |  |  | R: GGCAGAATTCTAGGGCCAGT |  |  |
| Sam_Hex1 | LT600695 | (AGAGGT)5 | F: GCAGTGGTGGAAGAGATTGC | 199-211 | NED ‡ |
|  |  |  | R: AAATTTGCATAGGGCAGCAC |  |  |
| Sam_Tri8 | LT600696 | (AAC)21 | F: AATCCCGACACAACCTCAAA | 100-127 | PET * |
|  |  |  | R: CGGTGGTAGAGCAAGTGAGG |  |  |

2. Departamento de Biodiversidad y Conservación, Real Jardín Botánico – CSIC, calle Claudio Moyano 1, 28014 Madrid, España.

**Corresponding author:** [priscila.rodriguez@ulpgc.es](mailto:priscila.rodriguez@ulpgc.es); +34928454543; ORCID: 0000-0002-7457-7596

Characteristics of the 7 microsatellite markers implemented for *Sambucus palmensis* in La Gomera. EMBL ID: Accession number per locus. Dye: Name of each fluorescent dye per locus. Multiplex; ‡Load A, *Load B. F: Forward. R: Reverse
