# Supplementary material for: The restoration of the endangered Sambucus palmensis after 30 years of conservation actions in the Garajonay National Park: genetic assessment and niche modeling
Source: PeerJ. 2018 Jun 12;6:e4985. doi: 10.7717/peerj.4985 (PMC6003393; doi:10.7717/peerj.4985)

The restoration of the endangered *Sambucus palmensis* after 30 years of conservation actions in the Garajonay National Park: genetic assessment and niche modelling

P. Rodríguez-Rodríguez[1], A. G. Fernández de Castro[2], P.A. Sosa[1]

1. Instituto Universitario de Estudios Ambientales y Recursos Naturales (IUNAT), Universidad de Las Palmas de Gran Canaria, Campus Universitario de Tafira, 35017 Las Palmas de Gran Canaria, España.

2. Departamento de Biodiversidad y Conservación, Real Jardín Botánico – CSIC, calle Claudio Moyano 1, 28014 Madrid, España.

Corresponding author: priscila.rodriguez@ulpgc.es; +34928454543; ORCID: 0000-0002-7457-7596

Online Resource 2. Output results from STRUCTURE HARVESTER. (A) The mean of log-likelihood values for each value of *K* (1-15), (B) Ad hoc statistic based on the rate of change in the log probability of data between successive *K* values (Δ*K*, following Evanno et al. (2005))

A)

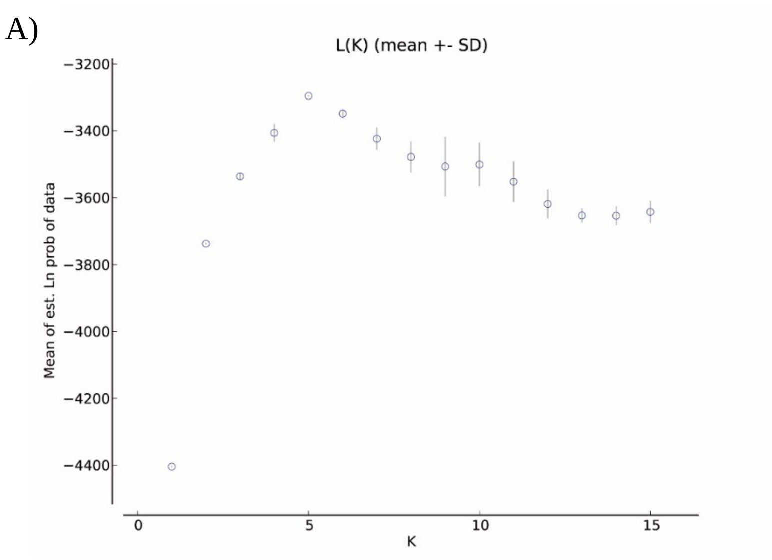

B)

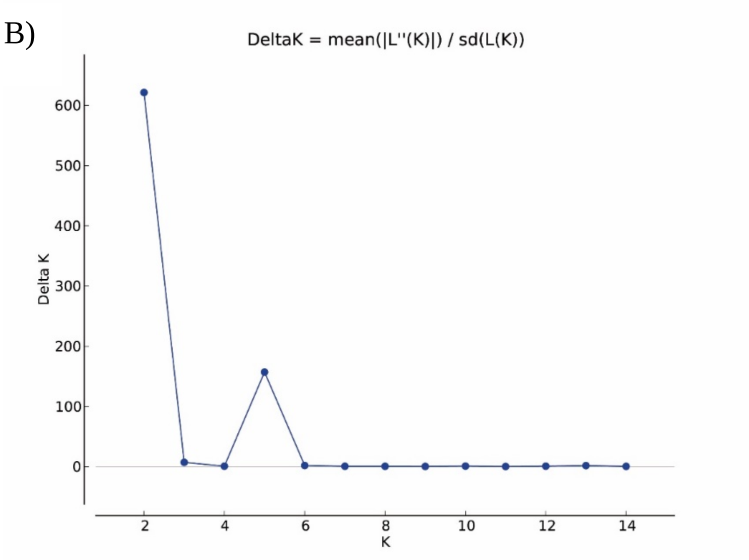

Supplement: Supplemental Information 2 — (A) The mean of log-likelihood values for each value of K (1–15), (B) Ad hoc statistic based on the rate of change in the log probability of data between successive K values (ΔK, following Evanno, Regnaut & Goudet (2005)). [file peerj-06-4985-s002.pdf]
