# Supplementary material for: The restoration of the endangered Sambucus palmensis after 30 years of conservation actions in the Garajonay National Park: genetic assessment and niche modeling
Source: PeerJ. 2018 Jun 12;6:e4985. doi: 10.7717/peerj.4985 (PMC6003393; doi:10.7717/peerj.4985)
Supplement: Supplemental Information 3 — Unique genotypes are assigned a numeric code and shared genotypes are coded with letters from A to ZZ. [file peerj-06-4985-s003.docx]

**The restoration of the endangered *Sambucus palmensis* after 30 years of conservation actions in the Garajonay National Park: genetic assessment and niche modelling**

**P. Rodríguez-Rodríguez^1^, A. G. Fernández de Castro^2^, P.A. Sosa^1^**

1. Instituto Universitario de Estudios Ambientales y Recursos Naturales (IUNAT), Universidad de Las Palmas de Gran Canaria, Campus Universitario de Tafira, 35017 Las Palmas de Gran Canaria, España.

2. Departamento de Biodiversidad y Conservación, Real Jardín Botánico – CSIC, calle Claudio Moyano 1, 28014 Madrid, España.

**Corresponding author:** [priscila.rodriguez@ulpgc.es](mailto:priscila.rodriguez@ulpgc.es); +34928454543; ORCID: 0000-0002-7457-7596

Genotypes per locality found for *Sambucus palmensis* in La Gomera. Unique genotypes are assigned a numeric code and shared genotypes are coded with letters from A to ZZ

| **Genotypes** | **ACE** | **CAN** | **ANG** | **CJO** | **CVA** | **CED** | **CHO** | **REJ** | **EPI** | **LIR** | **MER** | **MVA** | **PSA** | **PRO** | **RBR** | **Nº localities** | **Nº individuals** |
| --- | --- | --- | --- | --- | --- | --- | --- | --- | --- | --- | --- | --- | --- | --- | --- | --- | --- |
| **Unique** | **4** | **1** | **1** |  | **6** | **4** |  | **18** | **1** | **29** | **11** | **3** | **2** | **2** | **2** | 13 | **84** |
| A |  |  |  |  |  |  |  |  |  |  | 1 | 2 |  |  |  | 2 | 3 |
| AA |  |  |  |  |  | 1 |  |  | 1 |  |  |  |  |  |  | 2 | 2 |
| AAA | 1 |  |  |  |  |  |  | 1 |  |  |  |  |  |  |  | 2 | 2 |
| B |  | 1 |  |  |  |  |  | 3 |  |  |  |  |  |  |  | 2 | 4 |
| BB | 1 |  |  |  |  |  |  |  | 1 |  |  | 1 | 1 |  |  | 4 | 4 |
| BBB | 1 |  |  |  |  | 2 |  | 1 |  |  |  |  |  |  |  | 3 | 4 |
| C |  |  |  |  |  |  |  | 1 |  |  | 2 |  |  |  |  | 2 | 3 |
| CC |  |  |  |  |  | 2 |  |  |  |  |  |  |  |  |  | 1 | 2 |
| CCC | 2 |  |  |  |  | 1 |  | 1 |  |  |  |  |  |  |  | 3 | 4 |
| D |  |  |  |  |  |  |  | 2 |  |  |  |  |  |  |  | 1 | 2 |
| DD | 1 |  |  |  |  |  |  |  |  |  | 1 |  |  |  |  | 2 | 2 |
| DDD | 1 |  |  |  |  |  |  | 1 |  |  |  |  |  |  |  | 2 | 2 |
| E |  | 1 |  |  |  |  |  | 2 |  |  |  |  |  |  |  | 2 | 3 |
| EE | 1 |  |  |  |  | 1 |  |  |  |  | 1 |  |  |  |  | 3 | 3 |
| EEE | 1 |  |  |  | 1 | 1 |  |  |  |  |  |  |  |  |  | 3 | 3 |
| F |  |  |  |  |  |  |  | 2 |  |  |  |  |  |  |  | 1 | 2 |
| FF |  |  |  |  | 1 |  |  | 1 |  |  |  |  |  |  |  | 2 | 2 |
| FFF |  |  |  |  |  |  |  |  |  | 2 |  |  |  |  |  | 1 | 2 |
| G |  |  |  |  |  |  |  |  |  |  |  | 2 |  |  |  | 1 | 2 |
| GG |  |  |  |  |  |  |  | 1 | 1 |  |  |  |  |  |  | 2 | 2 |
| GGG |  |  |  |  |  |  |  |  |  | 2 |  |  |  |  |  | 1 | 2 |
| H |  |  |  |  | 1 |  |  | 1 | 1 |  |  |  |  |  |  | 3 | 3 |
| HH |  |  |  |  | 1 |  |  | 2 |  |  | 1 |  |  |  |  | 3 | 4 |
| HHH |  |  |  |  |  |  |  | 2 |  |  |  |  |  |  |  | 1 | 2 |
| I |  |  |  |  |  |  |  | 2 |  |  |  |  |  |  |  | 1 | 2 |
| II |  |  |  |  |  |  |  | 2 |  |  | 1 |  |  |  |  | 2 | 3 |
| III |  |  |  |  |  |  |  |  | 1 |  |  |  |  |  | 1 | 2 | 2 |
| J | 1 |  |  |  |  |  |  |  |  |  |  | 1 |  |  |  | 2 | 2 |
| JJ |  | 9 |  | 13 |  | 4 | 8 | 34 |  |  |  | 13 |  |  |  | 6 | 81 |
| JJJ |  |  |  |  |  | 1 |  | 1 | 1 |  |  | 2 |  |  |  | 4 | 5 |
| K |  |  |  |  | 1 |  |  |  |  |  |  | 2 |  |  |  | 2 | 3 |
| KK | 2 |  |  |  |  |  |  |  | 1 |  |  |  |  |  |  | 2 | 3 |
| KKK |  |  |  |  |  |  |  |  |  | 2 |  |  |  |  |  | 1 | 2 |
| L |  |  |  |  | 1 |  |  | 2 |  |  |  |  |  |  |  | 2 | 3 |
| LL | 1 |  |  |  | 1 |  |  | 1 |  |  |  |  |  |  |  | 3 | 3 |
| M |  |  |  |  |  |  |  | 2 |  |  |  |  |  |  |  | 1 | 2 |
| MM |  |  |  |  |  |  |  | 2 |  |  |  | 1 |  |  |  | 2 | 3 |
| N |  |  | 1 |  |  |  |  |  | 1 |  | 2 |  |  |  |  | 3 | 4 |
| NN |  |  |  |  |  | 2 |  | 2 | 1 |  |  |  |  |  |  | 3 | 5 |
| O |  |  |  |  | 1 |  |  | 1 |  |  |  |  |  |  |  | 2 | 2 |
| OO | 1 |  |  |  |  |  |  | 2 |  |  |  |  |  |  |  | 2 | 3 |
| P |  |  |  |  | 3 |  |  | 2 |  |  | 1 |  |  |  |  | 3 | 6 |
| PP | 1 |  |  |  |  |  |  | 3 |  | 1 | 2 |  |  |  |  | 4 | 7 |
| Q |  |  |  |  | 1 |  |  | 1 |  |  | 1 |  |  |  |  | 3 | 3 |
| QQ | 2 |  |  |  |  | 1 |  | 2 |  |  |  | 1 |  |  |  | 4 | 6 |
| R |  |  |  |  |  |  |  | 1 |  |  | 1 |  |  |  |  | 2 | 2 |
| RR |  |  |  |  |  |  |  |  |  | 2 |  |  |  |  |  | 1 | 2 |
| S | 1 |  |  |  |  | 1 |  | 1 |  |  |  | 1 | 1 |  |  | 5 | 5 |
| SS |  |  |  |  |  |  |  |  |  | 2 |  |  | 1 |  |  | 2 | 3 |
| T | 1 |  |  |  |  |  |  | 2 |  |  |  |  |  |  |  | 2 | 3 |
| TT | 2 |  |  |  |  | 5 |  | 1 |  |  |  | 1 |  |  |  | 4 | 9 |
| U |  |  |  |  | 1 | 1 |  |  |  |  |  |  |  |  |  | 2 | 2 |
| UU | 2 |  |  |  | 1 | 3 |  | 6 |  |  |  | 3 |  |  |  | 5 | 15 |
| V | 2 | 3 |  |  |  | 1 |  | 14 |  |  | 7 | 3 |  |  |  | 6 | 30 |
| VV |  |  |  |  |  | 1 |  | 2 |  |  |  |  |  |  |  | 2 | 3 |
| W |  |  |  |  |  | 2 |  | 1 |  |  | 2 |  |  |  |  | 3 | 5 |
| WW |  |  |  |  | 1 |  | 1 |  | 1 |  |  |  |  |  |  | 3 | 3 |
| X | 1 |  |  |  |  |  |  |  |  |  | 1 |  |  |  |  | 2 | 2 |
| XX |  |  | 1 |  |  |  |  | 1 | 1 |  |  | 1 |  |  |  | 4 | 4 |
| Y |  | 2 | 1 |  | 1 |  |  |  |  |  |  |  | 1 |  |  | 4 | 5 |
| YY | 1 |  | 1 |  | 1 |  |  |  |  |  |  |  | 1 |  |  | 4 | 4 |
| Z |  |  |  |  |  |  |  | 1 | 1 |  |  | 1 |  |  |  | 3 | 3 |
| ZZ |  |  |  |  | 2 |  |  | 1 |  |  | 1 |  |  |  |  | 3 | 4 |
| Nº of individuals | 31 | 17 | 5 | 13 | 25 | 34 | 9 | 129 | 13 | 40 | 36 | 38 | 7 | 2 | 3 |  | **402** |
| Nº of shared genotypes | 21 | 5 | 4 | 1 | 16 | 17 | 2 | 40 | 12 | 6 | 15 | 15 | 5 | 0 | 1 |  |  |
| Total genotypes | **25** | **6** | **5** | **1** | **22** | **21** | **2** | **58** | **13** | **35** | **26** | **18** | **7** | **2** | **3** |  |  |
