# Supplementary material for: The restoration of the endangered Sambucus palmensis after 30 years of conservation actions in the Garajonay National Park: genetic assessment and niche modeling
Source: PeerJ. 2018 Jun 12;6:e4985. doi: 10.7717/peerj.4985 (PMC6003393; doi:10.7717/peerj.4985)
Supplement: Supplemental Information 4 — Private alleles per locality are in bold and italics. Rare alleles (present in 4 localities or less) are in bold. [file peerj-06-4985-s004.docx]

**The restoration of the endangered *Sambucus palmensis* after 30 years of conservation actions in the Garajonay National Park: genetic assessment and niche modelling**

**P. Rodríguez-Rodríguez^1^, A. G. Fernández de Castro^2^, P.A. Sosa^1^**

1. Instituto Universitario de Estudios Ambientales y Recursos Naturales (IUNAT), Universidad de Las Palmas de Gran Canaria, Campus Universitario de Tafira, 35017 Las Palmas de Gran Canaria, España.

2. Departamento de Biodiversidad y Conservación, Real Jardín Botánico – CSIC, calle Claudio Moyano 1, 28014 Madrid, España.

**Corresponding author:** [priscila.rodriguez@ulpgc.es](mailto:priscila.rodriguez@ulpgc.es); +34928454543; ORCID: 0000-0002-7457-7596

Allele frequencies for the seven microsatellites studied in *Sambucus palmensis* localities in La Gomera. Private alleles per locality are in bold and italics. Rare alleles (present in 4 localities or less) are in bold

| **Locus** | **Allele** | **ACE** | **CAN** | **ANG** | **CJO** | **CVA** | **CED** | **CHO** | **REJ** | **EPI** | **LIR** | **MER** | **MVA** | **PSA** | **PRO** | **RBR** |
| --- | --- | --- | --- | --- | --- | --- | --- | --- | --- | --- | --- | --- | --- | --- | --- | --- |
| **Sam_Tet2** | **118** |  |  |  |  |  |  |  |  |  | **0.013** |  |  |  | **0.500** |  |
|  | **130** | 0.065 | 0.059 |  |  | 0.020 | 0.015 |  | 0.066 |  |  | 0.083 | 0.066 |  |  |  |
|  | **134** | 0.258 | 0.471 | 0.300 | 0.500 | 0.460 | 0.235 | 0.444 | 0.411 | 0.462 |  | 0.458 | 0.395 | 0.286 | 0.500 | 0.167 |
|  | **138** | 0.677 | 0.471 | 0.700 | 0.500 | 0.520 | 0.750 | 0.556 | 0.523 | 0.538 | 0.763 | 0.458 | 0.539 | 0.714 |  | 0.833 |
|  | **146** |  |  |  |  |  |  |  |  |  | ***0.225*** |  |  |  |  |  |
| **EMSn017** | **202** | 0.903 | 0.706 | 0.800 | 0.500 | 0.800 | 0.882 | 0.556 | 0.733 | 0.769 | 0.800 | 0.875 | 0.776 | 1.000 | 1.000 | 0.500 |
|  | **204** |  | **0.029** |  |  |  |  |  | **0.039** |  |  |  | **0.013** |  |  |  |
|  | **206** | 0.097 | 0.265 | 0.200 | 0.500 | 0.200 | 0.118 | 0.444 | 0.229 | 0.231 | 0.200 | 0.125 | 0.211 |  |  | 0.500 |
| **Sam_Hex1** | **199** | 0.597 | 0.824 | 0.600 | 1.000 | 0.640 | 0.574 | 0.944 | 0.767 | 0.615 | 1.000 | 0.708 | 0.737 | 0.714 | 0.500 | 0.667 |
|  | **211** | 0.403 | 0.176 | 0.400 |  | 0.360 | 0.426 | 0.056 | 0.233 | 0.385 |  | 0.292 | 0.263 | 0.286 | 0.500 | 0.333 |
| **Sam_Hex2** | **218** | 0.758 | 0.353 | 0.500 |  | 0.560 | 0.691 | 0.056 | 0.531 | 0.500 | 0.438 | 0.792 | 0.500 | 0.643 | 0.750 | 0.500 |
|  | **224** | 0.242 | 0.647 | 0.500 | 1.000 | 0.440 | 0.309 | 0.944 | 0.469 | 0.500 | 0.563 | 0.208 | 0.500 | 0.357 | 0.250 | 0.500 |
| **Sam_tri8** | **100** |  |  |  |  |  |  |  |  |  | ***0.225*** |  |  |  |  |  |
|  | **106** | **0.016** |  |  |  |  |  |  |  |  | **0.088** |  |  |  | **0.500** |  |
|  | **115** |  | **0.029** |  |  |  |  |  | **0.031** |  |  |  | **0.013** | **0.071** |  |  |
|  | **121** | 0.839 | 0.618 | 0.900 | 0.500 | 0.660 | 0.853 | 0.556 | 0.671 | 0.731 | 0.688 | 0.778 | 0.645 | 0.786 | 0.500 | 0.833 |
|  | **124** | 0.032 | 0.029 |  |  | 0.060 | 0.029 |  | 0.035 |  |  | 0.028 | 0.053 |  |  |  |
|  | **127** | 0.113 | 0.324 | 0.100 | 0.500 | 0.280 | 0.118 | 0.444 | 0.264 | 0.269 |  | 0.194 | 0.289 | 0.143 |  | 0.167 |
| **EMSn025** | **191** | 0.129 | 0.294 | 0.300 | 0.500 | 0.180 | 0.088 | 0.500 | 0.244 | 0.308 |  | 0.069 | 0.237 | 0.071 |  | 0.167 |
|  | **192** |  |  |  |  |  |  |  |  |  | ***0.175*** |  |  |  |  |  |
|  | **195** | 0.871 | 0.706 | 0.700 | 0.500 | 0.820 | 0.912 | 0.500 | 0.756 | 0.692 | 0.825 | 0.917 | 0.763 | 0.929 | 1.000 | 0.833 |
|  | **197** |  |  |  |  |  |  |  |  |  |  | ***0.014*** |  |  |  |  |
| **EMSn003** | **202** | 0.145 | 0.353 | 0.300 | 0.500 | 0.140 | 0.088 | 0.500 | 0.202 | 0.269 | 0.375 | 0.097 | 0.237 | 0.214 |  | 0.500 |
|  | **210** | 0.065 | 0.265 | 0.200 | 0.500 | 0.280 | 0.118 | 0.444 | 0.229 | 0.231 | 0.313 | 0.097 | 0.224 | 0.143 |  | 0.167 |
|  | **220** | 0.613 | 0.353 | 0.500 |  | 0.520 | 0.559 | 0.056 | 0.473 | 0.500 | 0.313 | 0.736 | 0.434 | 0.571 | 1.000 | 0.333 |
|  | **224** | 0.177 | 0.029 |  |  | 0.060 | 0.235 |  | 0.097 |  |  | 0.069 | 0.105 | 0.071 |  |  |
