# Supplementary material for: The restoration of the endangered Sambucus palmensis after 30 years of conservation actions in the Garajonay National Park: genetic assessment and niche modeling
Source: PeerJ. 2018 Jun 12;6:e4985. doi: 10.7717/peerj.4985 (PMC6003393; doi:10.7717/peerj.4985)
Supplement: Supplemental Information 5 — Individuals with unique or rare genotypes, which also presented a high individual heterozygosity and private or rare alleles were considered. ID = Code assigned to each individual, Genotype code = Identification code of the genotypes assigned to each individual, H-ind = Individual heterozygosity. [file peerj-06-4985-s005.docx]

**The restoration of the endangered *Sambucus palmensis* after 30 years of conservation actions in the Garajonay National Park: genetic assessment and niche modelling**

**P. Rodríguez-Rodríguez^1^, A. G. Fernández de Castro^2^, P.A. Sosa^1^**

1. Instituto Universitario de Estudios Ambientales y Recursos Naturales (IUNAT), Universidad de Las Palmas de Gran Canaria, Campus Universitario de Tafira, 35017 Las Palmas de Gran Canaria, España.

2. Departamento de Biodiversidad y Conservación, Real Jardín Botánico – CSIC, calle Claudio Moyano 1, 28014 Madrid, España.

**Corresponding author:** [priscila.rodriguez@ulpgc.es](mailto:priscila.rodriguez@ulpgc.es); +34928454543; ORCID: 0000-0002-7457-7596

List of the best candidates for future reintroduction programs. Individuals with unique or rare genotypes, which also presented a high individual heterozygosity and private or rare alleles were considered. ID = Code assigned to each individual, Genotype code = Identification code of the genotypes assigned to each individual, H-ind = Individual heterozygosity

| ID | Locality | | Genotype code | H-ind |
| --- | --- | --- | --- | --- |
| 732 | ACE | 25 | | 0.500 |
| 677 | ANC | B | | 0.625 |
| 890 | REJ | 8 | | 0.500 |
| 876 | REJ | 9 | | 0.750 |
| 885 | REJ | 18 | | 0.625 |
| 912 | REJ | 21 | | 0.625 |
| 935 | REJ | 41 | | 0.625 |
| 817 | LIR | 3 | | 0.750 |
| 811 | LIR | 50 | | 0.500 |
| 832 | LIR | 72 | | 0.500 |
| 823 | LIR | 74 | | 0.625 |
| 809 | LIR | 75 | | 0.500 |
| 837 | LIR | 76 | | 0.500 |
| 824 | LIR | 77 | | 0.500 |
| 827 | LIR | 78 | | 0.750 |
| 554 | LIR | 79 | | 0.750 |
| 806 | LIR | 80 | | 0.625 |
| 835 | LIR | 81 | | 0.625 |
| 802 | LIR | 83 | | 0.500 |
| 805 | LIR | 84 | | 0.500 |
| 813 | LIR | KKK | | 0.625 |
| 775 | MER | 43 | | 0.750 |
| 578 | MVA | 40 | | 0.750 |
| 590 | MVA | G | | 0.625 |
| 794 | PRO | 2 | | 0.500 |
