# Supplementary figures and images for: The restoration of the endangered Sambucus palmensis after 30 years of conservation actions in the Garajonay National Park: genetic assessment and niche modeling
Source: PeerJ. 2018 Jun 12;6:e4985. doi: 10.7717/peerj.4985 (PMC6003393; doi:10.7717/peerj.4985)

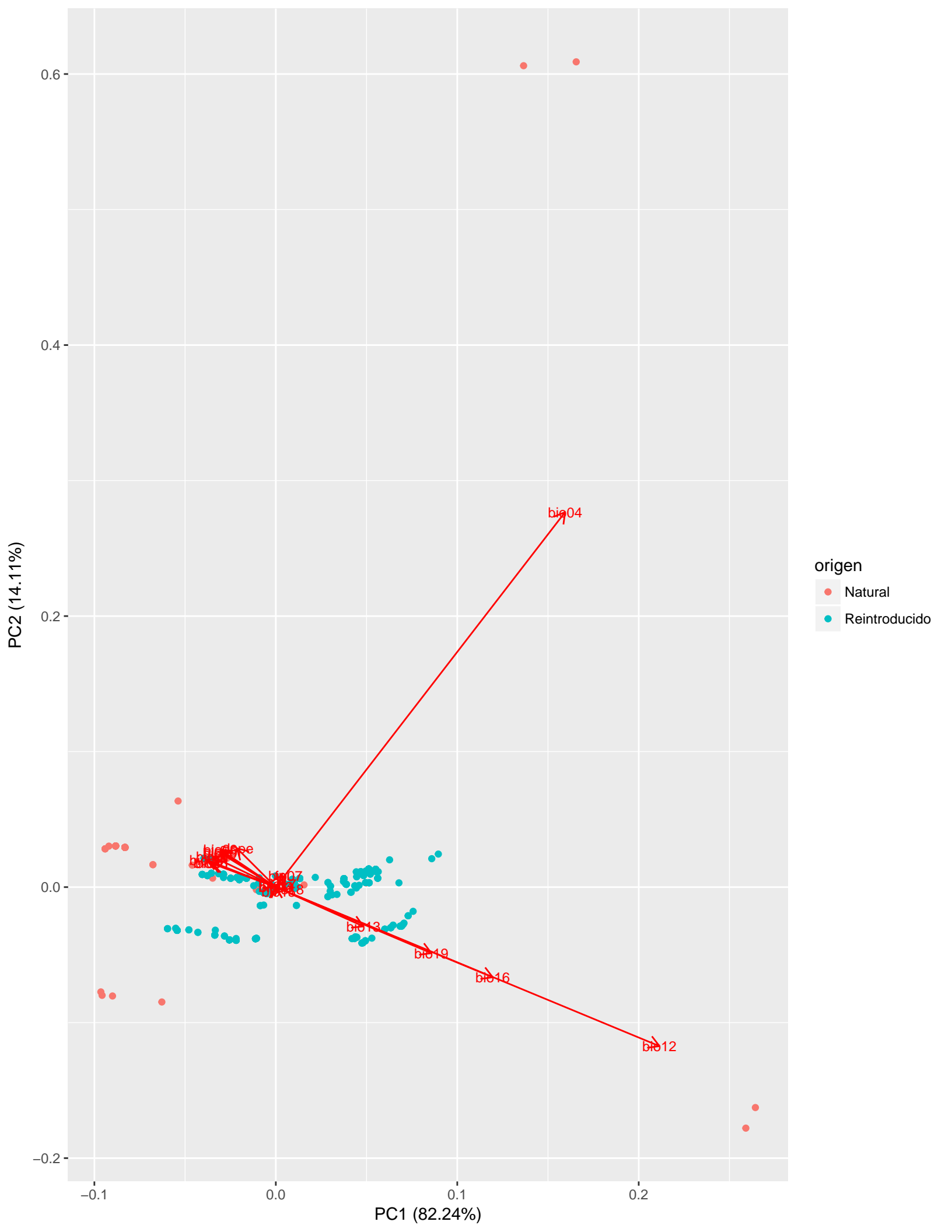

Supplement: Supplemental Information 6 [file peerj-06-4985-s006.pdf]

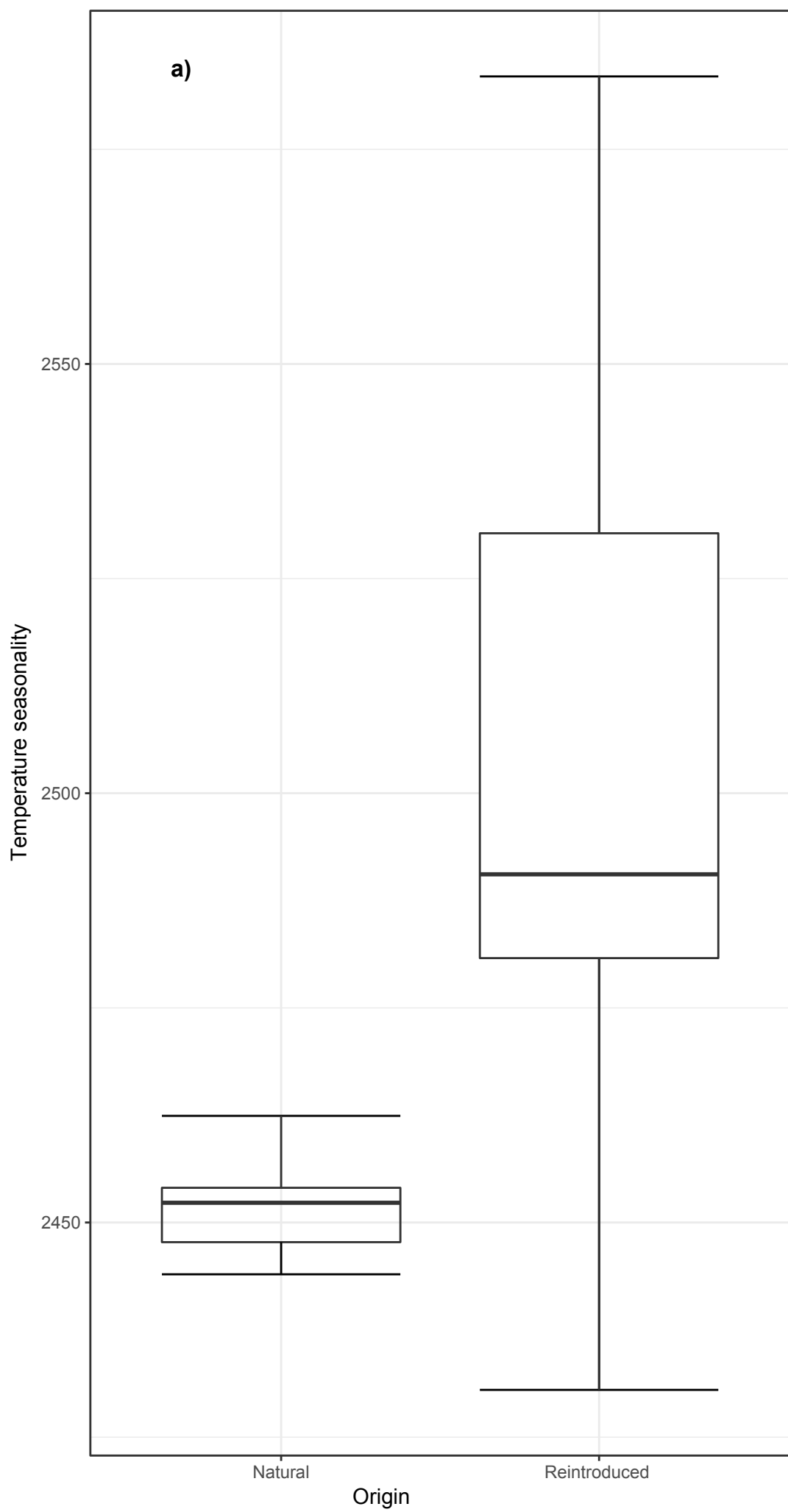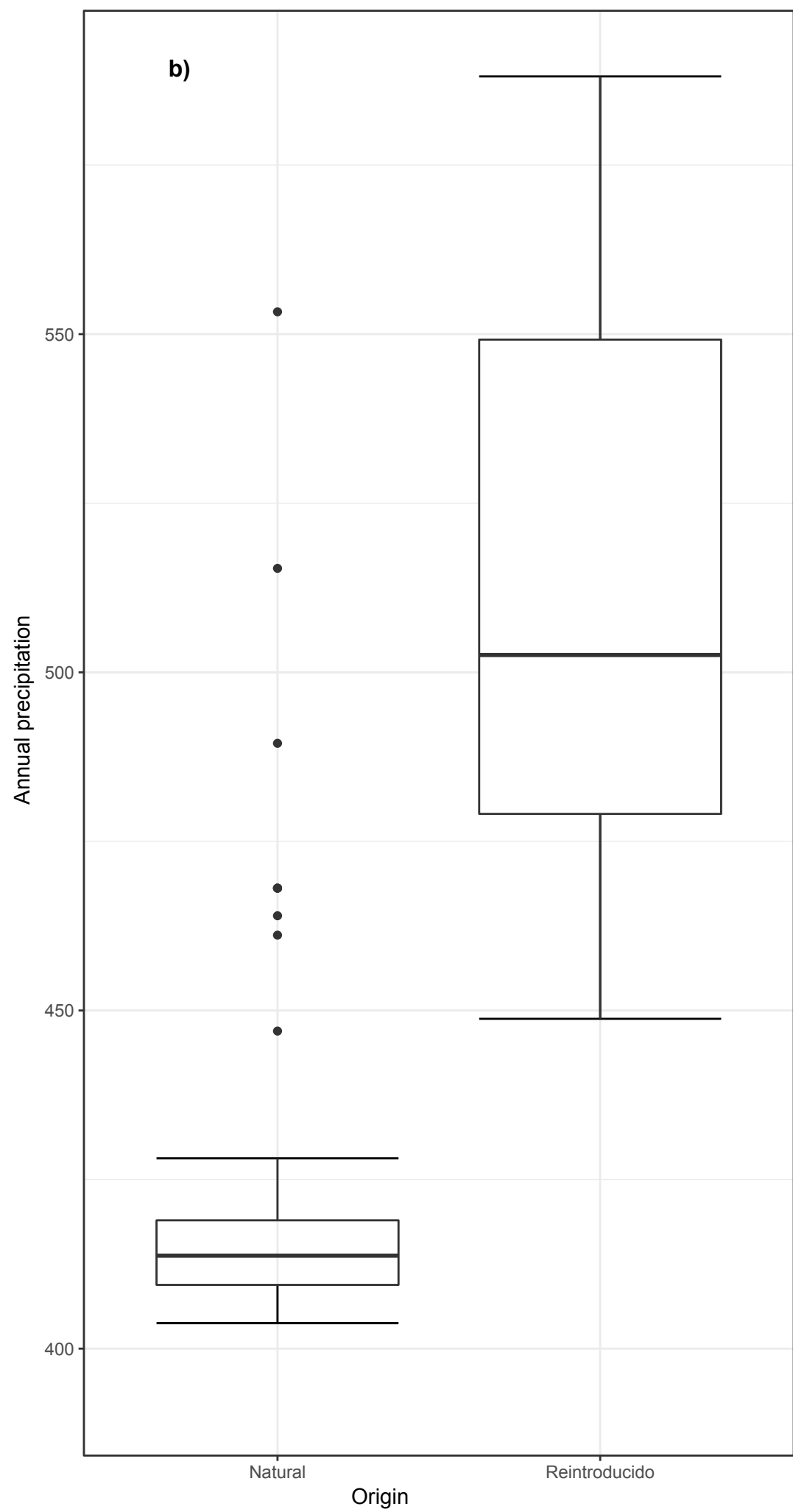

Supplement: Supplemental Information 7 — (A) Boxplots temperature seasonality and annual precipitation of natural occurrences and reintroduced occurrences. (B) Response curves for temperature seasonality and annual precipitation for the ensemble modelling calibrated with natural or reintroduced occurrences. [file peerj-06-4985-s007.pdf]

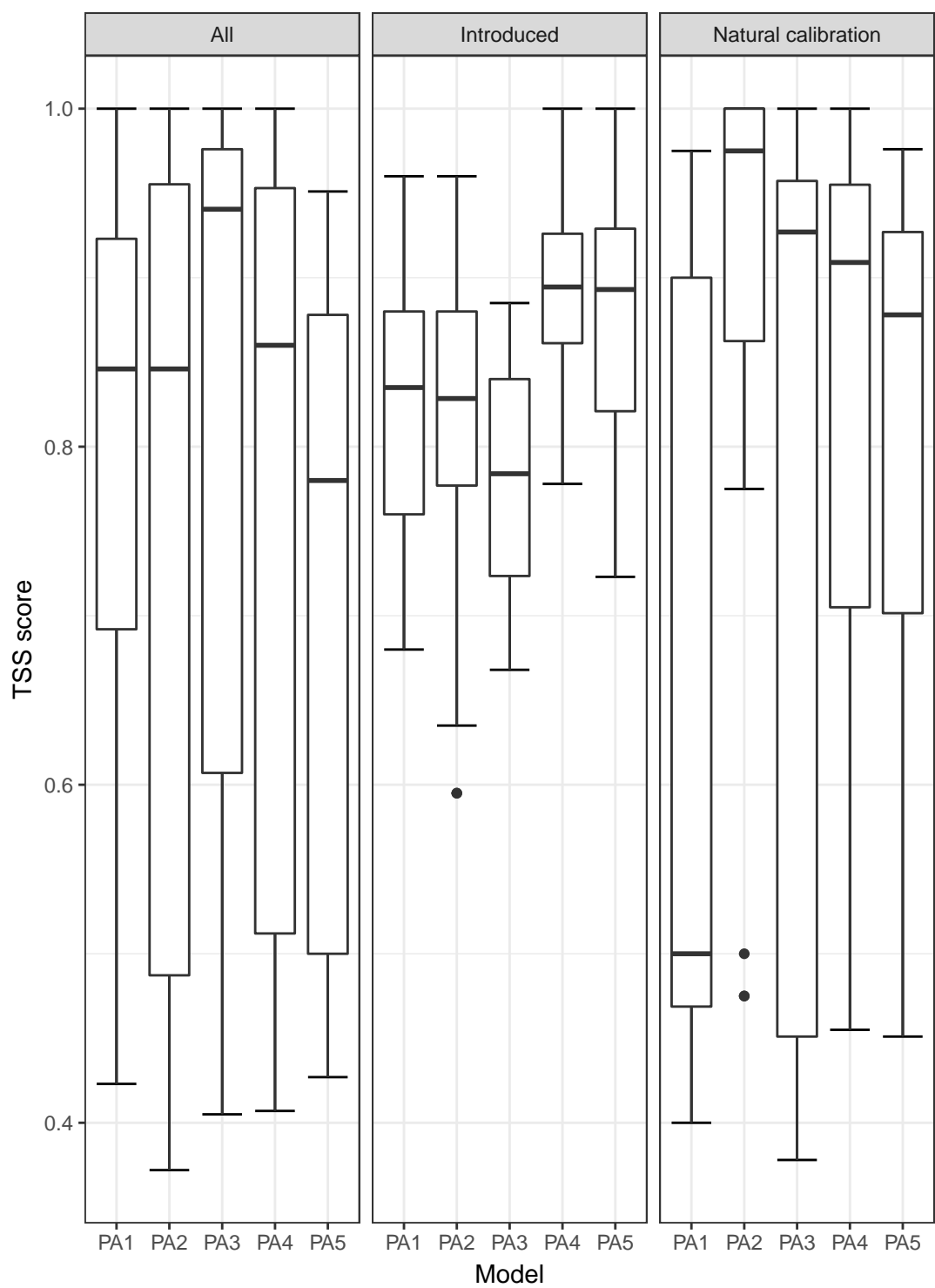

Supplement: Supplemental Information 8 [file peerj-06-4985-s008.pdf]
